# Students’ Perceptions of Psychiatry Placements and Absenteeism

**DOI:** 10.1192/bjo.2026.11351

**Published:** 2026-06-30

**Authors:** Linah Ahmed

**Affiliations:** Cumbria, Northumberland, Tyne, and Wear NHS Foundation Trust, Sunderland, United Kingdom. Central and Northwest London NHS Foundation Trust, Milton Keynes, United Kingdom

## Abstract

**Aims:**

The aim of this study was to explore medical students’ perceptions of psychiatry placements, with a particular focus on factors influencing engagement and absenteeism.Psychiatry placements represent a critical component of undergraduate medical education, yet concerns persist regarding inconsistent attendance and variable student engagement. This study sought to better understand the lived experiences of students during these placements in order to identify modifiable educational and structural factors. We hypothesised that stigma associated with psychiatry, the quality of the teaching environment, and the organisation and structure of placements significantly influence students’ attendance, enjoyment, and overall engagement during psychiatry rotations.

Psychiatry placements play an essential role in preparing future doctors to recognise, assess, and manage mental health conditions with competence and compassion. Despite this importance, psychiatry has historically been associated with stigma, both within society and the medical profession, which may negatively influence students’ attitudes and motivation. Reports of absenteeism and disengagement during psychiatry placements raise concerns about the effectiveness of current educational approaches. Gaining insight into students’ perspectives may help educators better understand barriers to engagement and inform improvements in placement design, teaching delivery, and student support.

**Methods:**

A qualitative study design was employed using semi-structured focus groups. Seven focus groups were conducted with medical students undertaking psychiatry placements, moderated by a Teaching Fellow to encourage open discussion. Topics explored included experiences of psychiatry placements, perceptions of the specialty, and factors influencing attendance or absenteeism. Audio recordings were transcribed verbatim, resulting in six usable transcripts. Data were analysed using reflexive thematic analysis, with coding and data management supported by Taguette software. The study was conducted with appropriate ethical approval and institutional governance oversight (Newcastle University; Application: 2524/30425).

**Results:**

Four key themes were identified: (1) stigma and misconceptions surrounding psychiatry, (2) teaching and learning environment, (3) drivers of absenteeism, and (4) factors promoting engagement. Stigma and limited understanding of psychiatry were found to negatively affect students’ motivation to attend placements. Positive teaching environments, supportive supervisors, and clear learning objectives were reported as strong facilitators of engagement. Absenteeism was commonly associated with perceptions of limited relevance to future practice, inconsistent teaching quality, and competing academic pressures.

**Conclusion:**

This study highlights the multifactorial drivers of engagement and absenteeism during psychiatry placements. Addressing stigma, improving teaching quality, and fostering supportive learning environments may enhance attendance and student experience. These findings support the development of targeted educational interventions aimed at improving engagement with psychiatry and nurturing confident, compassionate future clinicians.

